# Calcium-Sensing Receptor-Mediated Osteogenic and Early-Stage Neurogenic Differentiation in Umbilical Cord Matrix Mesenchymal Stem Cells from a Large Animal Model

**DOI:** 10.1371/journal.pone.0111533

**Published:** 2014-11-07

**Authors:** Nicola Antonio Martino, Stephan Joel Reshkin, Elena Ciani, Maria Elena Dell'Aquila

**Affiliations:** 1 Department of Emergency and Organ Transplantation (DETO) – Section of Veterinary Clinics and Animal Productions, University of Bari Aldo Moro, Valenzano, Bari, Italy; 2 Department of Biosciences, Biotechnologies and Biopharmaceutics (DBBB), University Campus “Ernesto Quagliariello” University of Bari Aldo Moro, Bari, Italy; National Cancer Institute, United States of America

## Abstract

**Background:**

Umbilical cord matrix mesenchymal stem cells (UCM-MSCs) present a wide range of potential therapeutical applications. The extracellular calcium-sensing receptor (CaSR) regulates physiological and pathological processes. We investigated, in a large animal model, the involvement of CaSR in triggering osteogenic and neurogenic differentiation of two size-sieved UCM-MSC lines, by using AMG641, a novel potent research calcimimetic acting as CaSR agonist.

**Methodology/Principal Findings:**

Large (>8µm in diameter) and small (<8µm) equine UCM-MSC lines were cultured in medium with high calcium (Ca^2+^) concentration ([Ca^2+^]_o_; 2.87 mM) and dose-response effects of AMG641 (0.01 to 3µM) on cell proliferation were evaluated. Both cell lines were then cultured in osteogenic or neurogenic differentiation medium containing: 1) low [Ca^2+^]_o_ (0.37 mM); 2) high [Ca^2+^]_o_ (2.87 mM); 3) AMG641 (0.05, 0.1 or 1 µM) with high [Ca^2+^]_o_ and 4) the CaSR antagonist NPS2390 (10 mM for 30 min) followed by incubation with AMG641 in high [Ca^2+^]_o_. Expression of osteogenic or neurogenic differentiation biomarkers was compared among groups. In both cell lines, AMG641 dose-dependently increased cell proliferation (up to P<0.001). Osteogenic molecular markers expression was differentially regulated by AMG641, with stimulatory (OPN up-regulation) in large or inhibitory (RUNX2 and OPN down-regulation) effects in small cells, respectively. AMG641 significantly increased alkaline phosphatase activity and calcium phosphate deposition in both cell lines. Following treatment with AMG641 during osteogenic differentiation, in both cell lines CaSR expression was inversely related to that of osteogenic markers and inhibition of CaSR by NPS2390 blocked AMG641-dependent responses. Early-stage neurogenic differentiation was promoted/triggered by AMG641 in both cell lines, as Nestin and CaSR mRNA transcription up-regulation were observed.

**Conclusions/Significance:**

Calcium- and AMG641-induced CaSR stimulation promoted *in vitro* proliferation and osteogenic and early-stage neurogenic differentiation of UCM-MSCs. CaSR activation may play a fundamental role in selecting specific differentiation checkpoints of these two differentiation routes, as related to cell commitment status.

## Introduction

Recent developments in stem cell biology research area have revealed that umbilical cord matrix (UCM, also known as Wharton's jelly) is a pivotal source of “young” mesenchymal stem cells (MSCs) considered as much more proliferative, immunosuppressive and even more therapeutically active than those from adult tissue sources [1]. Several groups reported success in isolating and establishing MSCs cultures from UCM in human [2]–[8] as well as in large animal models, such as horses [9]–[16], pigs [17], [18], and dogs [19]–[22].

The equine UCM (eUCM) is a well known source of MSCs that can be easily isolated, cryogenically preserved and *in vitro*-differentiated into adipocytes, chondrocytes, osteoblasts and in cells with a morphology typical of neurons with axon- and dendrite-like processes [9]-[12]. Moreover, eUCM-MSCs show expression of embryonic markers (Oct-4, SSEA-4, c-Myc), specific MSCs markers (CD44, CD90, CD105, and CD146) and lack expression of HLA-ABC, HLA1AG, and MHC II [12]. The low immunogenicity makes eUCM-MSCs ideal for cell therapy and regenerative medicine applications, both for species-specific purposes in equine medicine and as a large animal model of pre-clinical trials. The horse is of particular interest in the orthopaedic field, as bone, tendon and ligament diseases have a significant impact on equine industry. Also, enhancing research programs in this species could establish a particularly suitable animal model useful for pre-clinical trials in humans. Infact, current laboratory practices in equine regenerative medicine mirror those in the human field. Morever, the translational use of autologous and allogeneic MSCs for patient therapy far exceeds what is currently permitted in human medicine [23]. To date, stem cell therapy in equine orthopaedic diseases, a relatively new research area, has been based mainly on the use of bone marrow (BM-MSCs; [24], [25]), adipose tissue (Ad-MSCs; [26]), amnion-derived (AM-MSCs; [24]) and umbilical cord blood (UCB-MSCs; [27]). The use of stem cells in equine neurological diseases/neuropathies is still in a planning stage. The use of UCM-MSCs, due to their particularly advantageous features, such as ease of sourcing, high *in vitro* expandability and differentiation ability, immune-evasion and immune-regulation capacities, high homing ability, limited constraints due to ethical issues, low tumorigenicity, and even tumoricidal ability [1], [6], [28], [29] could allow significant improvements of clinical therapeutical applications.

An important procedural aspect of stem cell-based therapies is the control of proliferation and differentiation and extracellular calcium ion (Ca^2+^) is known as a potent mediator of the balance between proliferation and differentiation in a number of different cell types [30], [31]. The extracellular calcium-sensing receptor (CaSR) is a G protein–coupled receptor able to bind extracellular Ca^2+^ ions [32], firstly identified in bovine parathyroid cells by Brown et al., [33], and subsequently involved in the regulation of whole-body Ca^2+^ metabolism [30].

In this context, a large body of evidence supports a role of CaSR in cell proliferation [31], [34]–[41]. Indeed, a recent study from our unit reported the CaSR is expressed in eUCM-MSCs and is functionally active since calcium and the selective CaSR agonist NPS R-467 stimulate cell growth/proliferation in these cell lines, an effect which is reversed by the CaSR antagonist NPS2390 [13]. On the other hand, limited information is available on the role of CaSR in cell differentiation. Indeed, studies reported to date investigate its involvement in differentiation of specific lineages, such as osteoblasts [42], [43], osteoclasts [44], perinatal sympathetic neurons [45], epidermal initiation sites in mouse developing embryos and epidermic tissues [46] and preadipocytes [47] whereas only few studies reported its role in driving/regulating differentiation of embryonic [48] or fetal-derived stem cells ([49], for amniotic fluid-derived stem cells). No studies are reported to date on CaSR role in UCM-MSC differentiation.

Investigating whether CaSR affects ostegenic and neurogenic differentiation potency of UCM-derived MSCs by means of its selective agonists could contribute to elucidate differentiation mechanisms and to optimize differentiation protocols and the development of novel (even *in utero* and/or neonatal) targeted therapies in both bone diseases and neurodegenerative disorders. The aim of the present study was to investigate, in the horse as a large animal model, the involvement of CaSR on osteogenic and neurogenic differentiation potency of size-sieved UCM-MSC lines, by testing the *in vitro* effects of AMG641, a novel research calcimimetic acting as a CaSR agonist.

## Materials and Methods

### Chemicals

AMG641 (chemical name: (1R)-N- ((6-(methyloxy)-4′-(trifluoromethyl)-3-biphenylyl)methyl)-1-phenylethanamine;) was provided by Amgen Inc. (Thousand Oaks, California, USA; Research Program Agreement n° 2011568566). NPS2390

(Quinoxaline-2-carboxilic acid adamantan-1-ylamide) was purchased by Sigma (N4786), Milan Italy. NPS R-467 ((R)-N-(3-phenylpropyl)-a-methyl-3-methoxybenzylamide hydrochloride) was a kind gift of Dr. R. Caroppo (Dept. Biosciences, Biotechnologies and Biopharmaceutics, University of Bari, Italy)

### Cell lines isolation and stemness molecular charaterization

The study was carried out on two homogeneous subpopulations of equine UCM-MSCs (eUCM-MSCs) [12], [13]. Cells were isolated from perivascular Wharton's jelly by using a multi-dishes plate with transwell inserts of 8 µm pores [12]. Therefore, we have referred to the cells as size-sieved stem cells separated by their dimension, one larger than 8µm (namely, large cell line) and the other smaller than 8µm in diameter (namely, small cell line). The two cell lines were molecularly characterized and found as expressing CaSR at mRNA and protein level [13], pluripotency-specific antigens (OCT-4 and c-Myc), MSC markers (CD105, CD44, CD29 and CD166) and showing weak expression of antigens TRA-1-60 and SSEA-4 and lack of expression of the hematopoietic markers CD34, CD14 and MHC-II [12]. As well, in these cell lines, cell growth significantly increased in presence of high [Ca^2+^]_o_, (2.87 mM CaCl_2_), as previously reported [13].

### Culture conditions

Cells were cultured at 38.5°C in a humidified atmosphere (95%) under 5% CO_2_ in standard expansion medium, consisting of Dulbecco's Modified Eagle's Medium - High Glucose (DMEM) (Sigma D-5546) supplemented with 10% Fetal Calf Serum (FCS) (Sigma F3018), 100 U/ml penicillin, 100 µg/ml streptomycin, 0.25 µg/ml amphoterycin solution (Sigma A-5955), 2 mM L-glutamine (Sigma G-7513) and 10 ng/ml Epidermal Growth Factor (EGF; Sigma E-9644).

### Cell freezing

Standard medium supplemented with 10% (v/v) FCS and 10% (v/v) dimethyl sulfoxide (DMSO, Sigma D-5879) was used to cryopreserve cells in cryotubes that were stored at −80°C and then transferred to cryogenic containers with liquid nitrogen until molecular analysis.

### Osteogenic and neurogenic differentiation induction

Osteogenic and neurogenic *in vitro* differentiation potential was induced as previously reported [21], [50]. Cells at P3 were seeded at a density of 3000 cells /cm^2^ in six-well plates and were cultured until they reached approximately 80–90% confluence. For differentiation of MSCs into osteogenic lineage, cells were cultured for 21 days in medium consisting of HG-DMEM, supplemented with 10% FCS (Sigma), 100 U/ ml penicillin, 100µg/ml streptomycin, 0.25µg/ml amphotericin B, 2 mM L-glutamine, 10 mM beta-glycerophosphate, 0.1 µM dexamethasone and 250 µM ascorbic acid. Neurogenic induction was performed by culturing cells for 24 h in pre-induction medium consisting of HG-DMEM, 20% FCS and 1 mM beta-mercaptoethanol, with neural induction then performed by switching to a medium composed of DMEM plus 2% FCS, 2% dimethylsulphoxide (DMSO) and 200 mM butylated hydroxyanisole for 3 days. Non-induced control cells (negative controls) were cultured for the same length of time in standard medium (HG-DMEM supplemented with 10% FCS, 100 U/ ml penicillin, 100 µg/ml streptomycin, 0.25µg/ml amphotericin B, 2 mM L-glutamine).

### Osteogenic differentiation assessment

Osteogenesis was assessed by alkaline phosphatase (ALP) assay [51] and von Kossa staining [50] and by evaluating the relative abundance of the transcripts of two osteogenic differentiation markers, such as Runt-related transcription factor 2 (RUNX2) and Osteopontin (OPN). Briefly, ALP activity, a typical osteoblast marker, was assessed by using the Alkaline Phosphatase Kit (Sigma Aldrich), a commercial kit based on naphthol AS-BI and fast red violet LB [51]. After treatments in the conditions described below (Experiment 2), cells were fixed with a citrate-acetone-formaldehyde fixative for 30 sec at room temperature. After being rinsed with distilled water, cells were incubated for 15 min in dark with alkaline-dye mixture (NaNO2, FRV-Alkaline Solution, Naphthol AS-BI Alkaline Solution) and washed with distilled water. Cells were counterstained with hematoxylin solution, washed in tap water and evaluated under light microscopy. For von Kossa staining, cells were washed with PBS and then fixed with 10% formalin for 1 h at room temperature. The formalin was removed and cells were washed with distilled water. A 5% (w/v) silver nitrate solution was added and cells were exposed to UV light for 20 min. Reaction was stopped by using a 5% sodium thiosulphate solution for 2 min. Cells were washed with distilled water. Calcium phosphate deposits stained black [50].

### Neurogenic differentiation assessment

Neurogenic differentiation was assessed by conventional Nissl staining, beta III tubulin immunostaining and by evaluating the relative abundance of the transcripts of the two neurogenic differentiation markers, Glial Fibrillary Acidic Protein (GFAP) and Nestin. Briefly, for Nissl staining, cells were washed with PBS and then fixed with 10% formalin for 15 min at room temperature. The formalin was removed and cells were washed with distilled water and incubated in a 0.1% cresyl violet solution for 20 min. Cells were washed with distilled water and observed under light microscopy for the evaluation of Nissl bodies [50]. For beta III tubulin, a specific neuronal marker, cells were seeded on cover glass slides at the density of 1000 cell/cm^2^. After treatments in the conditions described below (Experiment 2), cells were permeabilized with 0.03% Triton X-100 for 15 min at room temperature and then incubated for 10 min in 1% (w/v) Bovine Serum Albumin (BSA) in PBS (PBS-BSA). After that, cells were incubated overnight at room temperature with a 1∶1000 dilution of a primary rabbit polyclonal antibody against the beta III tubulin (ab18207; Abcam, Cambridge, UK) in PBS-BSA. Cells incubated overnight in PBS-BSA were used as negative (no primary antibody) controls. At the end of incubation, cells were washed in PBS-BSA and incubated for 2 h at room temperature with a fluorescein isothiocyanate (FITC)- conjugated goat anti-rabbit IgG-secondary antibody (ab6717, Abcam) diluted 1∶500 in PBS-BSA. After washing, cells were fixed in 2% para-formaldehyde solution in PBS, mounted on slides and observed under oil immersion (630x magnification) with a C1/TE2000-U Nikon confocal laser-scanning microscope, equipped with an Argon Ions 488 laser and an 495–519 (B2-A) nm excitation/emission filter.

### Total RNA extraction

The RNeasy kit (QIAGEN, Milan, Italy) was used to extract total RNA. After thawing, cells were washed in PBS and then disrupted in Lysis Buffer RLT and homogenized at room temperature. A 70% ethanol solution was then added to the lysate, creating conditions that promoted selective binding of RNA to the RNeasy membrane. The lysate was transferred to an RNeasy spin column and centrifuged for 15 sec at 10000 rpm. The column was washed twice with washing buffer and finally, the total RNA was eluted in 30 µl of RNase-free water and stored at −80°C. The total amount of RNA of each sample was measured using the BioPhotometer plus (Eppendorf, Milan, Italy).

### Reverse Transcription PCR

The High Capacity cDNA Reverse Transcription kit (4368814, LifeTechnologies, Monza, Italy) was used to convert RNA to cDNA. After thawing, 1 µg of total RNA of each sample was added to 2 µL 10X RT Buffer, 0.8 µL 25X dNTP Mix, 2 µL RT Random Primers, 1 µL M-MLV Reverse Transcriptase, 1 µLRNase Inhibitor and nuclease-free water for a total volume of 20 µl and then, the solution was mixed gently and centrifuged briefly. Reaction tubes were incubated at 10°C for 10 min, then at 37°C for 120 min and finally at 85°C for 5 min. cDNA was stored at −20°C until Real Time PCR analysis.

### Real Time PCR

Real Time PCR was performed by using Real Time TaqMan technology and analyzed on automated “StepOne System” (Applied Biosystem, Monza, Italy). All TaqMan equine primers and probes were purchased from Life Technologies. Life Technologies inventored gene assays were adopted for Hypoxanthine phosphoribosyltransferase 1 (HPRT1; Ec03470219_m1) used as endogenous control, GFAP (Ec03470056_m1) and RUNX2 (Ec03469741_m1). For the equine CaSR, OPN and Nestin genes, primers and probes were specifically designed in the present study across an exon-exon junction, using Primer Express 3.0 software (Applied Biosystem) based on NCBI *Equus caballus* available nucleotidic sequences. The oligonucleotide sequences of the above Real Time PCR primers and probes are shown in Table 1. The TaqMan MGB (minor groove-binder) hybridization probe was labeled with a reporter dye (6-carboxy-fluorescein, FAM) on the 5′ nucleotide and a quenching dye with NFQ (non fluorescent quencher) on the 3′ nucleotide where MGB hyper-stabilized duplexes with complementary DNA. Samples were run in duplicates on Microamp fast optical 48-well reaction plate (Life Technologies) where twenty-microliters reactions for each well contained: 10 µL TaqMan gene expression Master Mix 2X (Life Technologies), 1µL 800 nM Primers and 250 nM Probe, 1–4 µL cDNA and RNase Free H_2_O. Cycling parameters were: 2 min at 50°C, 10 min at 95°C followed by 40 cycles of 15 s at 95°C and 1 min at 60°C. Data were collected by using the StepOne Software (Applied Biosystem, Monza, Italy) and relative quantification was performed by using comparative method after determining the Ct (threshold cycle) values for the reference endogenous control (HPRT1) and the target gene in each sample sets, according to the 2^−ΔΔCt^ method as described by the manufacturer. Changes in mRNA expression levels were calculated after normalization to HPRT1. The program calculates the ΔCt and the ΔΔCt with the formulas below: ΔCt  =  Ct_Mean(HPRT1) – Ct_Mean(target gene); ΔΔCt  =  ΔCt – ΔCt_Mean, so that the gene expression level  = 2^−ΔΔCt^. Changes in gene expression were reported as percentage changes relative to controls.

**Table 1:** Oligonucleotide sequences used for Real Time-PCR analysis.

Markers	Forward Primer 5′→ 3′	Reverse Primer 5′→ 3′	Probe 5′→ 3′
Calcium-Sensing Receptor (CaSR)	CTTGGCAGGTCCTGAAGCA	TGGTTGTTATACCCCCTGGTC	CTACGGCACCTCAAC
Osteopontin (OPN)	CGCAGATCTGAAGACCAGTATCCT	TTGCTTTCCACAGGTGATGTG	TGCTACGGAAGAGGAC
Nestin (Nes)	ACTGAGAAGTTCCAGCTGGC	GGACATCTTGAGGTGTGCCA	GGGCCTGCAGAGCCAGATCGCCCAGG

## Experimental Design

### Experiment 1: Effects of AMG641 on *in vitro* proliferation of size-sieved eUCM-MSCs

To evaluate the optimal concentration of the calcimimetic, large and small eUCM-MSCs cells were seeded in six well plates and cultured in the standard medium supplemented with rising concentrations of AMG641 (0.01, 0.05, 0.1, 0.5, 1 and 3 µM) in presence of 2.5 mM CaCl_2_. Calcium concentration was selected on the basis of a previous study from our unit [13] in which 2.5 mM CaCl_2_ was considered as CaSR EC_50_ in both cell lines. External Ca^2+^ concentration ([Ca^2+^]_o_) in the present study, as in our previous study by Martino et al., [13] was actually either 0.37 (low [Ca^2+^]_o_) or 2.37 mMCa^2+^ (high [Ca^2+^]_o_). Infact, standard medium was supplemented with 10% FCS and, since average [Ca^2+^]_o_ in FCS was around 1.6 mg/dL, thus 3.7 mM [52], [Ca^2+^]_o_ in our standard medium (10% FCS) was around 0.37 mM. When additional 2.5 mMCa^2+^ was included, final [Ca^2+^]_o_ was 2.87 mM (0.37+2.5 = 2.87 mM). Control cells were cultured in medium with additional 2.5 mM CaCl_2_ in the absence of AMG641. Negative controls were referred to cells cultured in medium with low calcium concentration (0.37 mM CaCl_2_) whereas positive controls were referred to values observed in cells cultured in high calcium concentration and in presence of 3 µM NPS R-467. For DT calculation, cells were seeded, at the density of 1000 cell/cm^2^. The DT data were calculated by using the following formula: CD  =  ln(Nf/Ni)/ln2 and DT = CT/CD, where DT is the cell-doubling time, CD is the cell-doubling number, CT is the cell culture time, Nf is the final number of cells and Ni the initial number of cells. Cells of each well were detached by using 0.05% trypsin/0.02% EDTA in PBS and were counted, by dilution (1∶1) in Trypan blue, with Burker′s counting chamber. Cell counts were performed on days 4^th^ after the beginning of the treatments. Data were expressed as mean ± standard deviation of values obtained in three replicates. The Student's t-test was used to evaluate statistical significances and values with P<0.05 were considered as statistically different.

### Experiment 2: Effects of AMG641 on osteogenic and neurogenic differentiation potential of eUCM-MSCs

Based on the results of experiment 1, three concentrations of AMG641 (0.05, 0.1 and 1 µM AMG641), that were shown to significantly stimulate cell proliferation, were selected to test its effects on osteogenic (experiment 2a) and neurogenic (experiment 2b) differentiation potential of eUCM-MSCs.

Cells were cultured in following conditions: 1) control (CTRL): osteogenic or neurogenic differentiation medium, as described above; 2) high external calcium (2.5 mM CaCl_2_): differentiation medium +2.5 mM CaCl_2_; 3) agonist (2.5 mM CaCl_2_+ AMG641): differentiation medium plus 2.5 mMCaCl_2_ and AMG641 (0.05 or 0.1 or 1 µM); 4) antagonist/agonist (2.5 mM CaCl_2_ + NPS2390 + AMG641): standard differentiation medium plus 2.5 mM CaCl_2_ and 1 µM AMG641. In these samples, before CaSR agonist addition, cells were preincubated for 30 min with 10 mM NPS2390. In both cell lines, after 14 days in vitro culture under osteogenic differentiation induction conditions, ALP activity was analyzed and, after 21 days, von Kossa staining was performed and CaSR and osteogenic differentiation markers mRNA expression levels were evaluated. As well, in both cell lines, after 4 days of neurogenic differentiation treatments, Nissl staining and beta III tubulin immunostaining were performed and CaSR and neurogenic differentiation markers mRNA expression levels were evaluated. The Student's t-test was used to evaluate the statistical significance of the results. Within each differentiation lineage, differentiation biomarkers relative expression in the four above mentioned treatments groups (CTRL, high calcium, agonist and antagonist/agonist) were compared. ALP activity, extracellular matrix mineralization (calcium deposits), Nissl bodies formation and beta III tubulin immunostaining were quantified by densitometric analysis using Adobe Photoshop. For each experimental condition, areas of constant size were measured. Staining intensity was evaluated by recording: 1) the total stained area, 2) the number of stained spots and 3) the mean spot area. Data (mean ± standard deviation of values obtained in three replicates) were expressed as number of pixels. The Student's t-test was used to evaluate statistical significances and values with P<0.05 were considered as statistically different.

## Results

### Experiment 1: AMG641 stimulates *in vitro* proliferation of eUCM-MSCs

**Figure 1:**
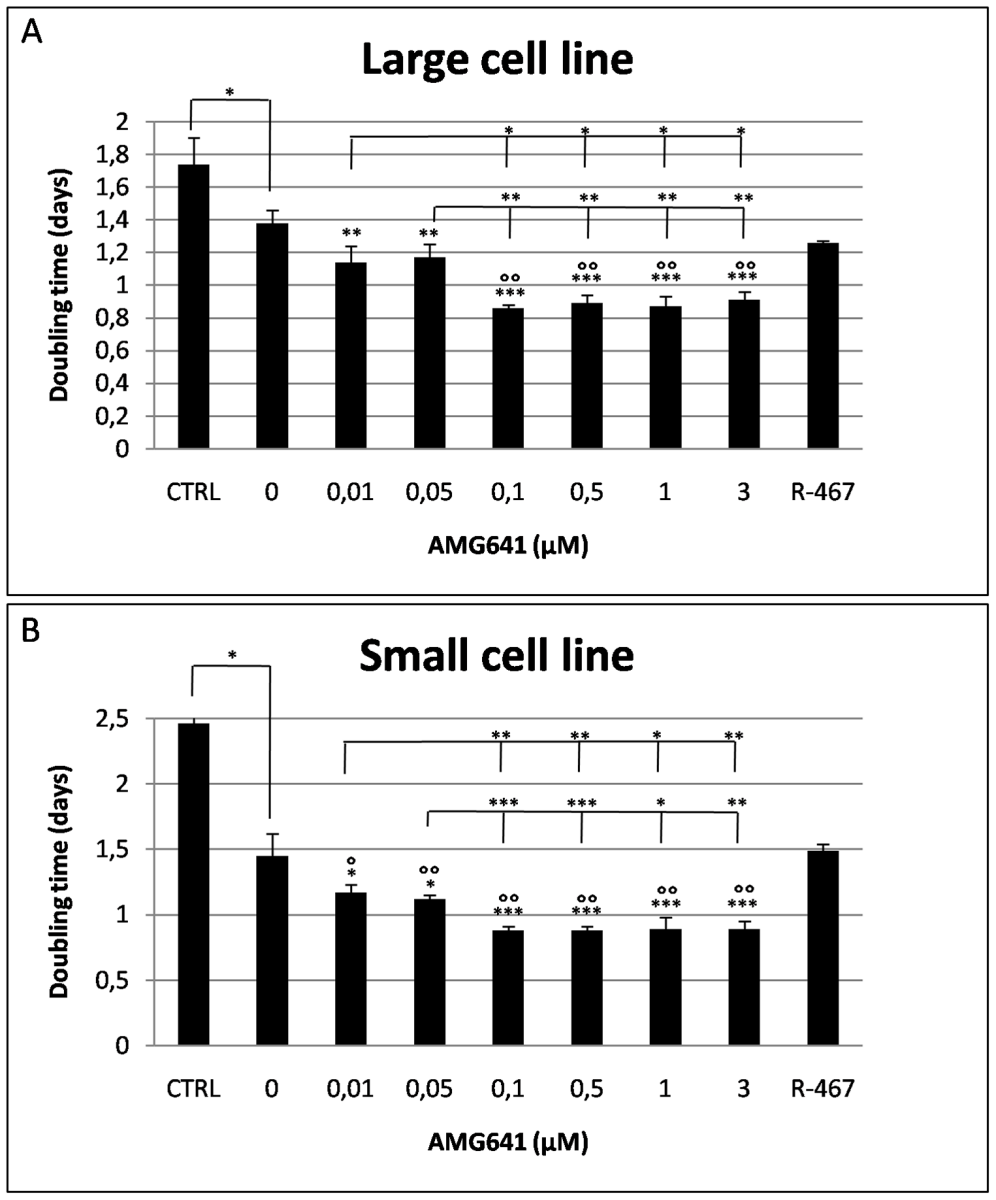
Effects of the calcimimetic AMG641 on *in vitro* proliferation of size-sieved eUCM-MSC lines. Cells were plated in 6-wells plates and treated as described in materials and methods. The effects of increasing doses of AMG641 in presence of 2.5 mM extracellular Ca^2+^ on the large (Panel A) and the small (Panel B) eUCM-MSC cell line are shown. In both cell lines, AMG641 significantly increased cell proliferation, expressed as significantly reduced doubling time (DT) values. DT values in cells cultured in medium with low calcium concentration (negative controls, CTRL) were significantly higher than those of cell cultured in high calcium (0 µM AMG641). DT values of cells cultured in presence of NPS R-467 (3µM, positive controls) were significantly higher that those of AMG641-treated cells. Data are mean ± standard deviation of values obtained in three replicates. Student's t-test: comparisons AMG641-treated cells vs controls (low and high calcium) and among used AMG641 concentrations: *P<0.05; **P<0.01; ***P<0.001; comparisons AMG641- vs NPS R-467-treated cells: °P<0.01; °°P<0.001.

**Figure 2:**
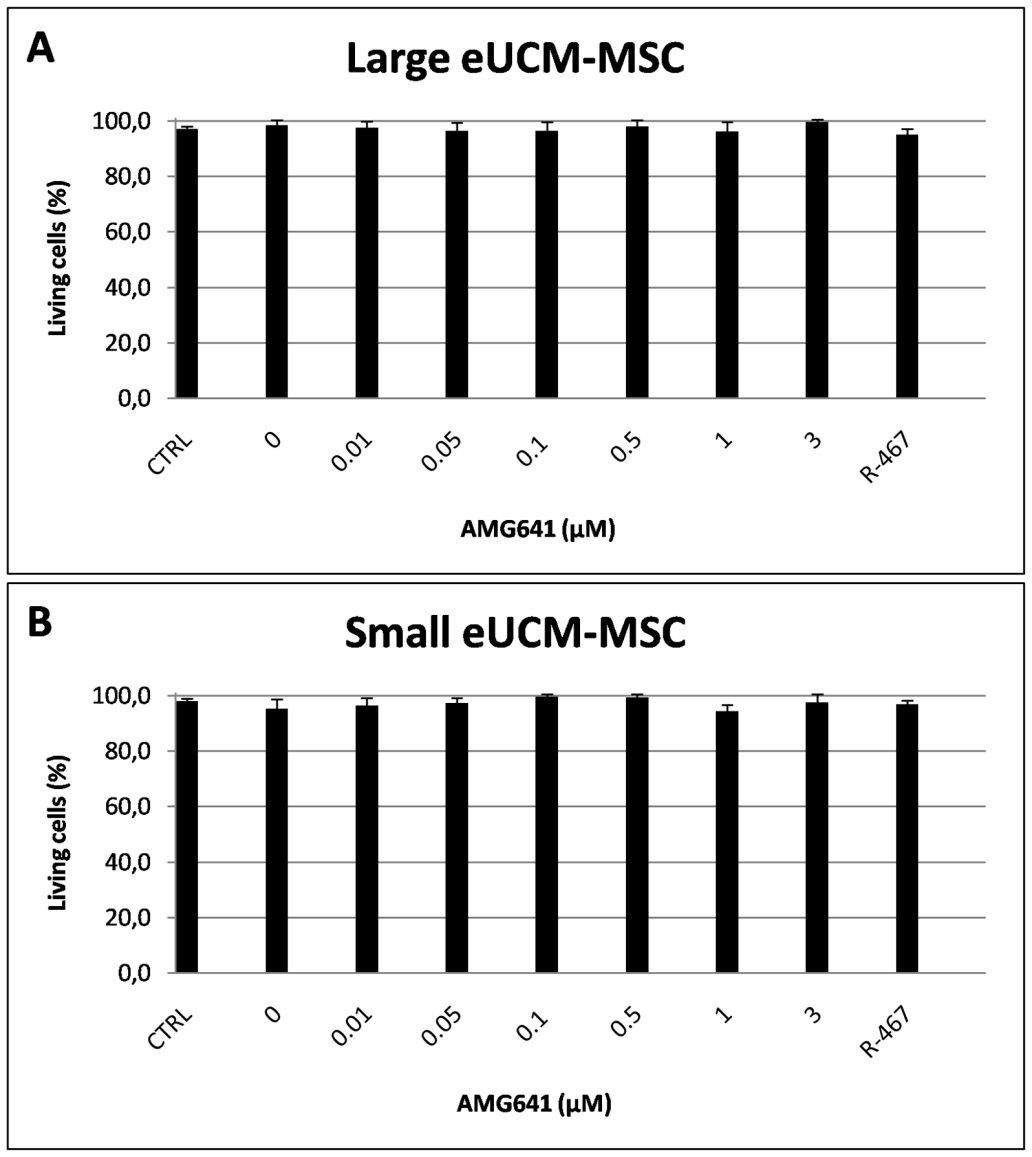
Effects of the calcimimetic AMG641 on cell viability of size-sieved eUCM-MSC lines. Cell viability is expressed as percentage of living (unstained) cells after Trypan blue staining. The effects of increasing doses of AMG641 in presence of 2.5 mM extracellular Ca^2+^ on the large (Panel A) and the small (Panel B) eUCM-MSC cell line are shown. Cell viability remained at high values, indicating non toxic effects of AMG641 on these cell lines. As in Figure 1, values in cells cultured in medium with low calcium concentration are referred as negative controls (CTRL) and values observed in cells cultured in presence of 3 µM NPS R-467 are referred as positive controls (R-467). Data are mean ± standard deviation of values obtained in three replicates. Student's t-test: NS.

In both cell lines, the proliferation rate was significantly increased upon AMG641 treatment and the effects of this compound were very similar between the two cell lines. In detail, in the large cell line, AMG641 significantly reduced DT values compared with controls (P<0.01 in cells treated with the two lowest AMG641 concentrations, i.e. 0.01 and 0.05µM AMG641; P<0.001 in cells treated with the four remaining higher AMG641 concentrations, i.e. 0.1, 0.5, 1 and 3 µM AMG641; Fig. 1, Panel A). Likewise, in the small cell line, AMG641 significantly reduced DT values compared with controls (P<0.05 in cells treated with the two lowest AMG641 concentrations; P<0.001 in cells treated with the remaining higher AMG641 concentrations; Fig. 1, Panel B). In both cell lines, DT values in cells cultured in medium with low calcium concentration (negative controls, CTRL) were significantly higher than that of cell cultured in high calcium (P<0.05). As well, in both cell types, DT values of cells cultured in presence of 3 µM NPS R-467 (positive controls) were significantly higher that those of 0.1 to 3 µM AMG641-treated cells (P<0.001 in both large and small cells), indicating a more sustained action of AMG641 than NPS R-467. In both cell lines, treatment with AMG641 had no effect on cell viability (Fig. 2; Panel A and Panel B), indicating non toxic effect of this compound on these cell lines.

### Experiment 2a: CaSR activation promotes osteogenic differentiation of eUCM-MSCs

#### Effects of AMG641 on bone-like cell morphology and histochemical features

**Figure 3:**
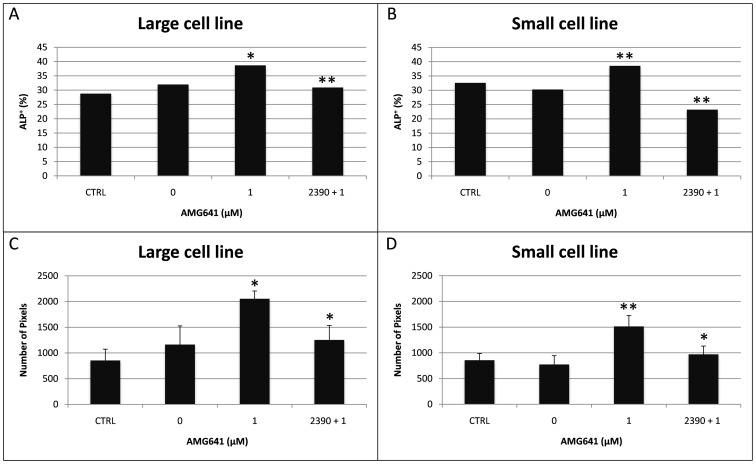
Effects of calcium and the calcimimetic AMG641 on osteogenic differentiation efficiency of size-sieved eUCM-MSC lines, as assessed by ALP activity. Panel A: percentages of ALP+ cells in the large and small cell lines observed after treatments, as described in M&M, Experiment 2: (CTRL) control cells differentiated *in vitro* in presence of low [Ca^2+^]_o_; (0) cells differentiated *in vitro* in presence of high [Ca^2+^]_o_; (1) AMG641-treated cells (treated with 1 µM AMG641 in presence of of high [Ca^2+^]_o_); (NPS2390+1) NPS2390/AMG641-treated cells (cells pre-incubated for 10 min in 10 mM NPS2390 and then cultured in 1 µM AMG641in presence of of high [Ca^2+^]_o_). A minimum of 500 cells per experimental condition was counted. In both cell lines, the addition of AMG641 significantly increased the proportion of ALP+ cells (1 vs 0: P<0.05 and P<0.01 for large and small cell lines, respectively). As well, in both cell lines, the CaSR antagonist reversed the stimulatory effect of AMG641 on ALP activity (P<0.01). Panel B: Quantification analysis was performed by measuring the total stained area normalized on the number of counted cells. Data are expressed as number of pixels. Significantly higher ALP activity was found after AMG641 treatment (1 vs 0: P<0.05) and significantly lower ALP activity was found after antagonist treatment compared with AMG641 (NPS2390+1 vs 1: P<0.05). Non-induced controls (non induced to differentiate in vitro) showed 0 value in both cell lines and in both panels, as ALP positivity was not observed at all in these samples (data not shown).

**Figure 4:**
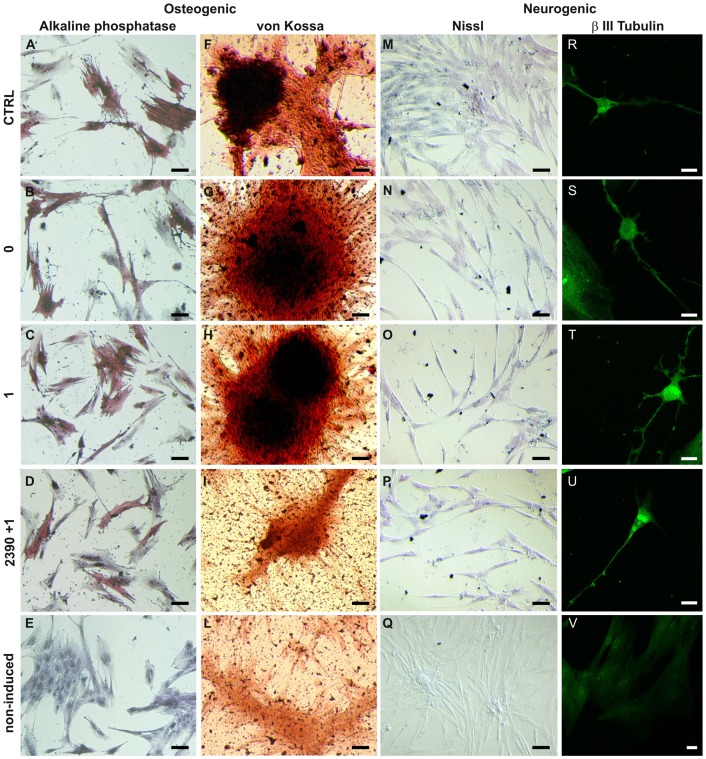
Bone-like and neuron-like morphology, cytochemical and histochemical features of AMG641-treated large eUCM-MSCs as evaluated by ALP activity, von Kossa and Nissl stainings and beta III tubulin immunodetection. Photomicrographs representative of the morphological appearance of the large eUCM-MSC line induced to differentiate *in vitro* toward osteogenic (1^st^ and 2^nd^ column) or neurogenic (3^rd^ and 4^4h^ column) lineages in presence of 1µM AMG641 and observed after ALP activity assay, von Kossa and Nissl stainings, and beta III tubulin immunostaining. (A, F, M, R) control cells differentiated *in vitro* in presence of low [Ca^2+^]_o_; (B, G, N, S) cells differentiated *in vitro* in presence of high [Ca^2+^]_o_; (C, H, O, T) AMG641-treated cells (treated with 1 µM AMG641 in presence of of high [Ca^2+^]_o_); (D, I, P, U) NPS2390/AMG641-treated cells (cells pre-incubated for 10 min in 10 mM NPS2390 and then cultured in 1 µM AMG641in presence of of high [Ca^2+^]_o_); (E, L, Q, V) non-induced controls. Osteogenic differentiation was evidenced by increased ALP activity observed after 14 days *in vitro* culture and by the formation of mineralized matrix as shown by von Kossa staining after 21 days *in vitro* culture. No apparent differences can be seen for ALP activity and for the presence of mineral deposits after treatment with high [Ca^2+^]_o_ (B, G) or with AMG641 in high [Ca^2+^]_o_ (C, H) compared with controls (CTRL: A, F). ALP positivity and amount of calcium phosphate deposits appear to be reduced in antagonist/agonist-treated cells (D, I) compared with those treated with the CaSR agonist AMG641 (C, H). Neurogenic differentiation was evidenced by the presence of Nissl bodies and beta III tubulin positivity, as observed after 4 days *in vitro* culture. All cells directed toward neurogenic differentiation showed diffused discrete granular bodies after Nissl staining with no apparent differences among those cultured in presence of low (M) or high [Ca^2+^]_o_ (N) or high [Ca^2+^]_o_ plus AMG641 (O) or in cells pre-incubated with the CaSR antagonist NPS2390 and subsequently treated with AMG641 (P). Some cells showed neuron-like appearance and pancytoplasmic beta III tubulin immunopositivity in all induced conditions (R,S,T, U) with no apparent differences among treatments. Non-induced control cells were unstained for osteogenic (E, L) and neurogenic (Q, V) markers. Black scale bars indicate 10 µm; white scale bars indicate 5 µm.

**Figure 5:**
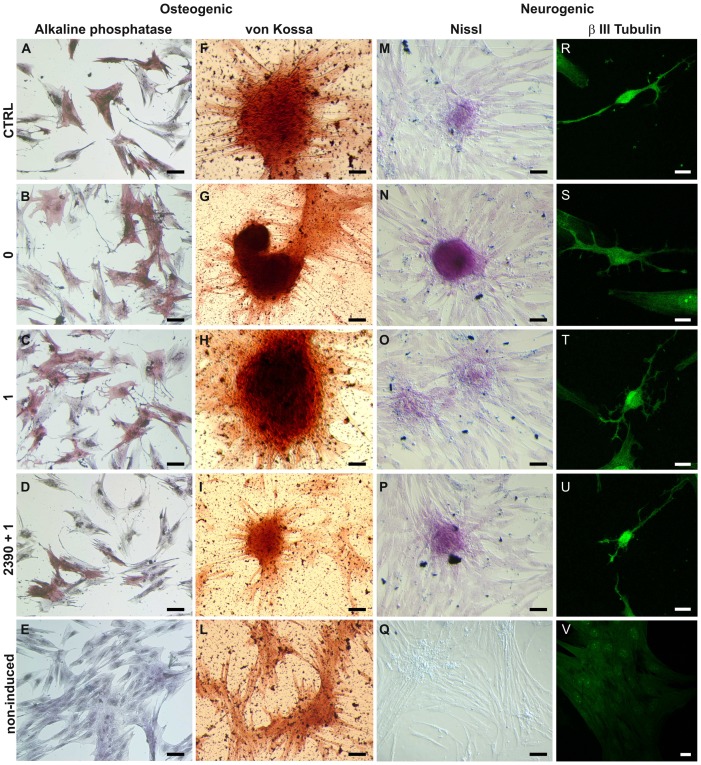
Bone-like and neuron-like morphology, cytochemical and histochemical features of AMG641-treated small eUCM-MSCs as evaluated by ALP activity, von Kossa and Nissl stainings and beta III tubulin immunodetection. Photomicrographs representative of morphological appearance of the small eUCM-MSC line induced to differentiate *in vitro* toward osteogenic (1^st^ and 2^nd^ column) or neurogenic (3^rd^ and 4^4h^ column) lineages in presence of 1µM AMG641 and observed after ALP activity assay, von Kossa and Nissl stainings, and beta III tubulin immunostaining. As in Figure 4, control cells differentiated *in vitro* in the presence of low [Ca^2+^]_o_ (A, F, M, R), cells differentiated *in vitro* in the presence of high [Ca^2+^]_o_ (B, G, N, S), AMG641-treated cells (treated with 1 µM AMG641 in the presence of high [Ca^2+^]_o_) (C, H, O, T), NPS2390/AMG641-treated cells (cells pre-incubated for 10 min in 10 mM NPS2390 and then cultured in 1 µM AMG641in the presence of high [Ca^2+^]_o_) (D, I, P, U) and non-induced controls (E, L, Q, V) are shown. As in the large cells, no apparent differences can be seen for ALP activity and for the presence of mineral deposits after treatment with high [Ca^2+^]_o_ (B, G) or with AMG641 in high [Ca^2+^]_o_ (C, H) compared with controls (CTRL: A, F). Even in small cells, ALP positivity and the amount of calcium phosphate deposits appears to be significantly reduced in antagonist/agonist-treated cells (D, I) compared with those treated with the CaSR agonist AMG641 (C, H). All cells directed toward neurogenic differentiation showed discrete granular bodies after Nissl staining no apparent differences among those cultured in presence of low (M) or high [Ca^2+^]_o_ (N) or high [Ca^2+^]_o_ plus AMG641 (O) or in cells pre-incubated with the CaSR antagonist NPS2390 and subsequently treated with AMG641 (P) can be seen. Some cells showed neuron-like appearance as they displayed beta III tubulin immunopositivity throughout the cell body, the axon and dendritic-like extensions with no apparent differences among treatments. Non-induced control cells were unstained for osteogenic (E, L) and neurogenic (Q, V) markers. Black scale bars indicate 10 µm; white scale bars indicate 5 µm.

**Figure 6:**
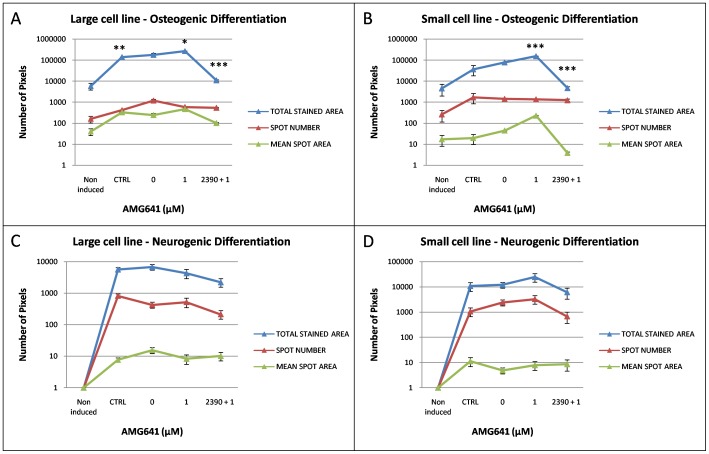
Effects of calcium and the calcimimetic AMG641 on osteogenic and neurogenic differentiation efficiency of size-sieved eUCM-MSC lines, as assessed by quantification of extracellular matrix mineralization and Nissl bodies formation. Densitometric analysis was performed in large and small cells after von Kossa (Panel A and B) and Nissl staining (Panel C and D). For each experimental condition, areas of constant size were measured. Staining intensity was evaluated by recording: 1) the total stained area, 2) the number of stained spots and 3) the mean spot area. Data (mean ± standard deviation of values obtained in three replicates) are expressed as number of pixels. Cells induced to osteogenic differentiation showed higher mineralization levels in response to CaSR activation by the calcimimetic AMG641 (large and small cells). The addition of the CaSR antagonist NPS2390 reversed the agonists effects. In both cell lines, no statistical differences were noticed among treatments for Nissl staining intensity values. Student's t-test: comparisons AMG641-treated cells vs controls (low and high calcium) and among used AMG641 concentrations: *P<0.05; **P<0.01; ***P<0.001.

As a first approach to evaluate the effects of higher Ca^2+^ on AMG641 action on osteogenic differentiation potency of eUCM-MSCs lines, cell morphology, cytochemical and histochemical characteristics were analyzed. Cells were evaluated at P3. In both cell lines, ALP positivity was found in cells induced to differentiate through the osteogenic lineage with significantly higher proportions of ALP+ cells after treatment with high [Ca^2+^]_o_ plus AMG641 compared with high [Ca^2+^]_o_ alone (P<0.05 and P<0.01 for the large and the small cell lines, respectively). As well, in both cell lines, the CaSR antagonist NPS2390 reversed the stimulatory effect of AMG641 (P<0.01; Fig. 3, panel A, B). ALP quantification analysis confirmed these observations, as significantly higher ALP activity was found in AMG641-treated cells compared with high [Ca^2+^]_o_ alone controls (P<0.05) and this effect was reversed by addition of NPS2390 (P<0.05; Fig. 3, panel C, D). Figures 4 and 5 show representative photomicrographs of ALP and von Kossa stained cells in both large and small cell lines, respectively. Clearly evident ALP positivity and conspicuous mineralized extracellular matrix deposition by von Kossa staining with no apparent differences among those cultured in presence of low (Fig. 4 A, F) or high [Ca^2+^]_o_ (Fig. 4 B, G) or high [Ca^2+^]_o_ plus AMG641 (Fig. 4 C, H) were observed. Instead, in cells pre-incubated with the CaSR antagonist NPS2390 and subsequently treated with AMG641, reduced proportion of ALP+ cells and reduced amount of calcium phosphate deposits were observed (Fig. 4 D, I). Non-induced negative control cell lines showed neither ALP positivity nor relevant calcium phosphate deposits after von Kossa staining (Fig. 4 E, L). These observations can be similarly referred to the large, as well as to the small cell line (Fig. 5 A to L). Quantification analysis of von Kossa stained specimens allowed to observe that cells induced to osteogenic differentiation showed significantly higher mineralization level in response to CaSR activation by the calcimimetic AMG641 (comparison 1 µM AMG641-treated cells vs high high [Ca^2+^]_o_ control: P<0.05 in large cells, Fig. 6, Panel A; P<0.001 in small cells, Fig. 6, Panel B). The addition of the CaSR antagonist NPS2390 significantly reversed the agonists effects (P<0.001 in both large and small cells).

#### Effects of high [Ca^2+^]_o_ on CaSR and osteogenic biomarkers expression

**Figure 7:**
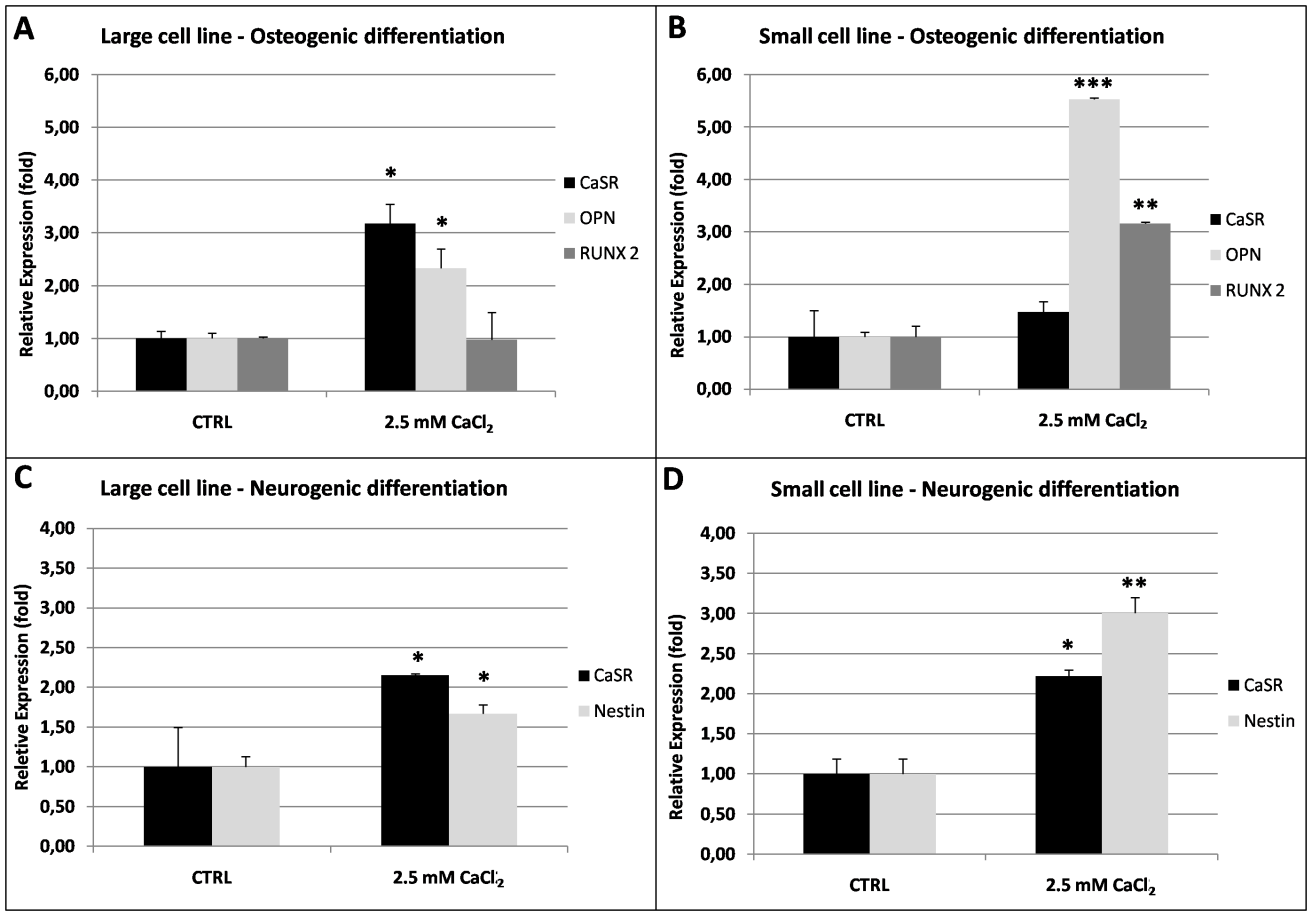
Relative abundance of CaSR and specific osteogenic and neurogenic differentiation biomarkers in eUCM-MSCs upon Ca^2+^–induced CaSR stimulation. Panels A, B: Quantitative Real Time RT-PCR analysis of CaSR, OPN and RUNX2 transcripts in high [Ca^2+^]_o_-treated equine UCM-MSCs (2.5 mM CaCl_2_) versus low [Ca^2+^]_o_ controls (CTRL). In the large cell line, additional Ca^2+^ up-regulated CaSR (P<0.05) and OPN (P<0.05) mRNA expression even if it had no effect on RUNX2 (Panel A). In the small cell line, high [Ca^2+^]_o_ significantly increased both OPN and RUNX2 transcripts level (P<0.001 and P<0.01, respectively; Panel B) but it had no effects on CaSR expression. Panels C, D: Quantitative Real Time RT-PCR analysis of CaSR, Nestin and GFAP transcripts in high [Ca^2+^]_o_-treated equine UCM-MSCs (2.5 mM CaCl_2_) versus low [Ca^2+^]_o_controls (CTRL). In both cell lines, additional Ca^2+^ up-regulated Nestin (P<0.05 in the large cell line, Panel C; P<0.01 in the small cell line, Panel D) and CaSR (P<0.05) mRNA expression whereas the GFAP transcript was not detected. For each sample, average Ct data (mean±SD of three independent experiments in duplicate) were normalized relatively to the abundance of HPRT1 mRNA (endogenous control) and normalized values were compared between groups. Student's t-Test; *P<0.05; **P<0.01; ***P<0.001.

Elevating [Ca^2+^]_o_ in culture medium during osteogenic differentiation significantly increased the relative abundance of osteogenic differentiation biomarkers compared with that observed in standard differentiation conditions (CTRL). In the large cell line, additional Ca^2+^ up-regulated Osteopontin (OPN, P<0.05) and CaSR (P<0.05) mRNA expression even if it had no effect on relative expression of the Runt-related transcription factor, RUNX2 (Fig. 7, Panel A) whereas in the small cell line, high [Ca^2+^]_o_ significantly increased both OPN and RUNX2 transcripts level (P<0.001 and P<0.01, respectively; Fig. 7, Panel B) but it had no effects on CaSR expression.

#### Effects of AMG641 on CaSR and osteogenic biomarkers expression

**Figure 8:**
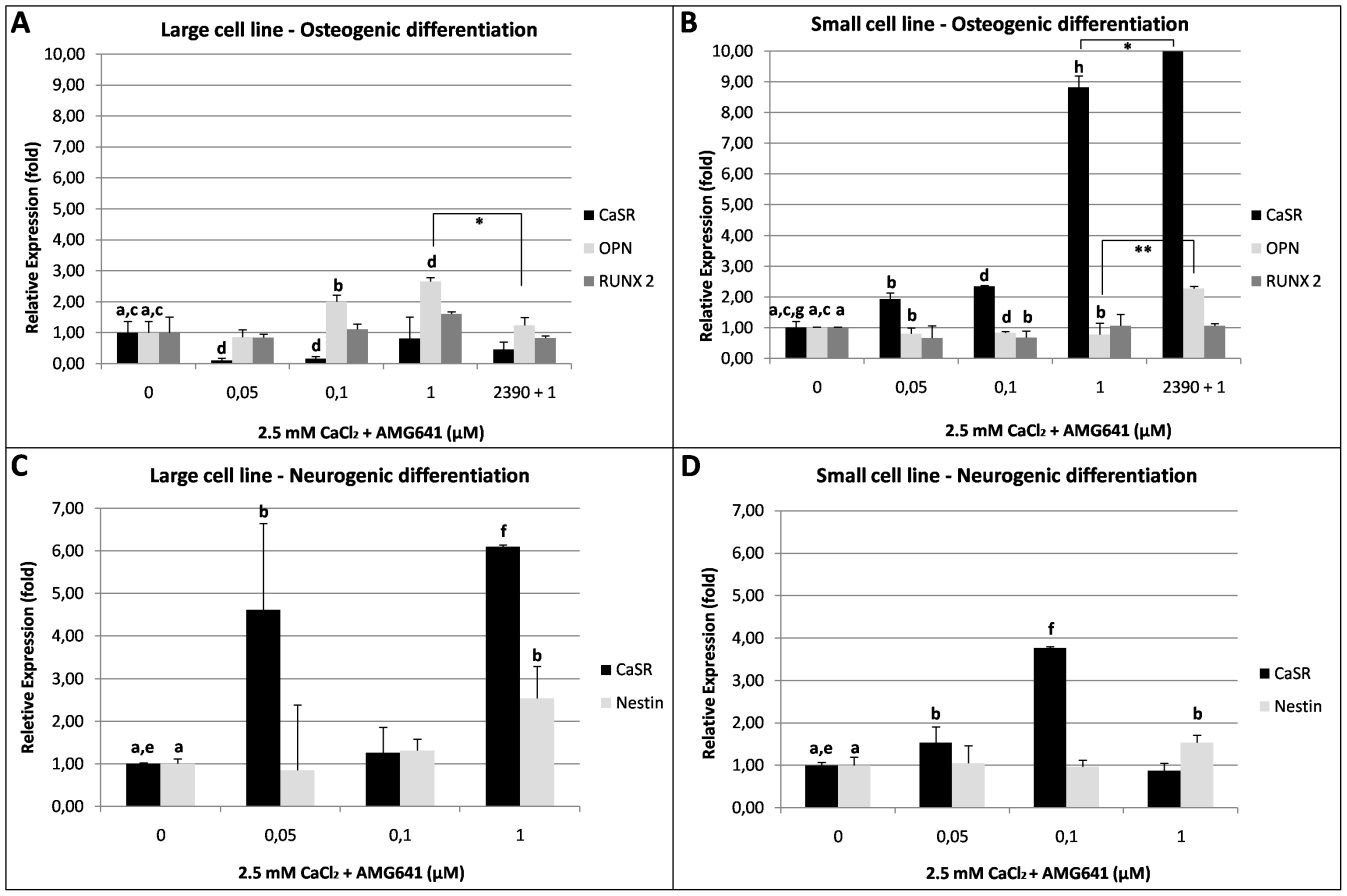
Relative abundance of CaSR and specific osteogenic and neurogenic differentiation biomarkers in eUCM-MSCs upon calcimimetic AMG641-induced CaSR stimulation. Panels A, B: Quantitative Real Time RT-PCR analysis of CaSR, OPN and RUNX2 transcripts inhigh [Ca^2+^]_o_ and (0.05, 0.1 or 1 µM) calcimimetic AMG641-treated equine UCM-MSCs (2.5 mM CaCl_2_+AMG641) or cells pre-incubated with the CaSR antagonist NPS2390 (2.5 mM CaCl_2_+NPS2390+AMG641) versus cells cultured in high [Ca^2+^]_o_ (2.5 mM CaCl_2_) alone (indicated as 0 µM AMG641). In the large cell line, AMG641 increased OPN transcription (P<0.05 and P<0.01 with 0.1 and 1 µM AMG641, respectively) and had no effect on RUNX2 expression (Panel A). In the small cell line, AMG641 significantly down-regulated OPN (P<0.05, P<0.01 and P<0.05 respectively in cells stimulated with 0.05, 0.1 and 1 µM AMG641) and RUNX2 transcription (P<0.05 in cells treated with 0.1 µM AMG641) (Panel B). Upon AMG641 addition, CaSR expression was significantly down-regulated in the large cell line (P<0.01 at 0.05 and 0.1 µM AMG641), but markedly up-regulated (up to approximately 9 folds) in the small cell line, with a dose-response effect (P<0.05, P<0.01 and P<0.0001, at 0.05, 0.1 and 1 µM AMG641, respectively). In both cell lines, the addion of NPS2390 reversed the effects of AMG641 on OPN mRNA levels, with significant reduction in large cells (P<0.01) and significant increase in small cells (P<0.001) whereas it had no effects on RUNX2 mRNA levels. In both cell lines, the CaSR relative abundance remained at comparable levels after agonist or antagonist/agonist treatments. Panels C, D: Quantitative Real Time RT-PCR analysis of CaSR, Nestin and GFAP transcripts in cells cultured in the conditions as described for Panels A,B. In both cell lines, AMG641 significantly increased Nestin mRNA level at the highest tested dose (1 µM, P<0.05; Panels C and D). The GFAP transcript was never detected in any examined condition (not shown). As well, in both cell lines, AMG641 significantly increased CaSR transcription level (up to six folds in the large cell line; P<0.05 and P<0.001 at 0.05 and 1 µM AMG641 respectively; Panel C and up to approximately 4 folds in the small cell line; P<0.05 an P<0.001 at 0.05 and 0.1 µM AMG641, respectively; Panel D). In both cell line types, no repeatable reverse effects were obtained upon NPS2390 addition (data not shown). For each sample, average Ct data (mean±SD of three independent experiments in duplicate) were normalized relatively to the abundance of HPRT1 mRNA (endogenous control) and normalized values were compared among groups. Student's t-Test: a,b P<0.05; c,d P<0.01; e,f P<0.001; * P<0.01; ** P<0.001.

The addition of AMG641 in presence of high [Ca^2+^]_o_ during osteogenic differentiation induced opposite effects in the two cell lines. In the large cell line, AMG641 increased OPN transcription (P<0.05 and P<0.01 with 0.1 and 1 µM AMG641, respectively) and had no effect on RUNX2 expression (Fig. 8, Panel A) while, conversely, in the small cell line, AMG641 significantly down-regulated OPN (P<0.05, P<0.01 and P<0.05 in cells stimulated with 0.05, 0.1 and 1 µM AMG641, respectively) and RUNX2 transcription (P<0.05 in cells treated with 0.1 µM AMG641) (Fig. 8, Panel B). Interestingly, CaSR transcription was found to be inversely related to the expression of osteogenic markers. In fact, it was significantly down-regulated in the large cell line (P<0.01 at 0.05 and 0.1 µM AMG641), but markedly up-regulated in the small cell line, with a clear dose-response effect (P<0.05, P<0.01 and P<0.0001, at 0.05, 0.1 and 1 µM AMG641, respectively). In both cell lines, the addition of NPS2390 reversed the effects of the CaSR agonist AMG641 on OPN mRNA levels, with significant down-regulation in large cells (P<0.01) and up-regulation in small cells (P<0.001) whereas it had no effects on RUNX2 mRNA levels. In both cell lines, NPS2390 was not able to reverse the effect of AMG641 on CaSR relative abundance.

### Experiment 2b: CaSR activation promotes neurogenic differentiation of eUCM-MSCs

#### Effects of AMG641 on neuron-like cell morphology

The effects of AMG641 in presence of additional Ca^2+^ on neurogenic differentiation potency of eUCM-MSC lines, were assessed by cell morphology and cytochemical characteristics. Cells were evaluated at P3. Cells directed toward neurogenic differentiation showed diffused discrete granular bodies after Nissl staining, with no apparent differences among those cultured in presence of low (Fig. 4 M) or high [Ca^2+^]_o_ (Fig. 4 N) or high [Ca^2+^]_o_ plus AMG641 (Fig. 4 O) or in cells pre-incubated with the CaSR antagonist NPS2390 and subsequently treated with AMG641 (Fig. 4 P). A low percentage of cells (around 1%) became more elongated and showed neuronal-like morphology, as they displayed beta III tubulin immunopositivity throughout the cell body, the axon and dendritic-like extensions (Fig. 4 R,S,T,U). Non-induced negative controls showed neither Nissl nor beta III tubulin labeling (Fig. 4 Q, V). These observations can be similarly referred to the small cell line (Fig. 5 M to V). In both cell lines, densitometric analysis for Nissl bodies quantification did not show statistically significant differences among groups (Fig.6, Panel C and Panel D). Densitometric analysis of beta III tubulin labeled cells did not reveal statistical differences among groups (data not shown).

#### Effects of high [Ca^2+^]_o_ on CaSR and neurogenic biomarkers expression

In both cell lines, additional Ca^2+^ up-regulated Nestin (P<0.05 in the large cell line, Fig. 7, Panel C; P<0.01 in the small cell line, Fig. 7, Panel D) and CaSR (P<0.05 in both cell lines) mRNA expression whereas the GFAP transcript was never detected in any examined culture condition.

#### Effects of AMG641 on CaSR and neurogenic biomarkers expression

The addition of AMG641 during neurogenic differentiation induced similar effects in the two cell lines. In detail, in both cell lines, it significantly increased Nestin mRNA level when added at 1 µM (P<0.05; Fig. 8 Panels C and Ds). The GFAP transcript was never detected in both cell lines, either cultured with or without AMG641. As well, in both cell lines, the treatment with AMG641 significantly increased the CaSR transcript level (P<0.05 and P<0.001 at 0.05 and 1 µM AMG641 respectively in the large cell line; Fig. 8, Panel C and P<0.05 an P<0.001 at 0.05 and 0.1 µM AMG641, respectively in the small cell line; Fig. 8, Panel D). No repeatable reverse effects were obtained upon NPS2390 addition.

## Discussion

The proliferation study was performed with the aim to preliminary testing *in vitro* AMG641 activity on equine fetal adnexa-derived size-sieved UCM-MSCs. A dose-response effect of AMG641 on the proliferation rate of eUCM-MSCs was observed. AMG641 in presence of high [Ca^2+^]_o_ significantly increased cell proliferation in both large and small eUCM-MSCs lines. To the best of our knowledge, this is the first study on the effects of AMG641 on cell proliferation of *in vitro* cultured cell lines and, more specifically, in fetal-adnexa derived mesenchymal stem cell lines. AMG641 is a research calcimimetic mainly reported to date in studies on calcium homeostasis disorders [54]–[57]. Recent studies reported its effect as selective activator of CaSR [55], [58]–[61]. Only one study has been reported to date, on the effects of AMG641 on *in vivo* cell proliferation [58] and reduced or unchanged proliferation rates were observed in cells obtained from parathyroid tissues of five-sixth nephrectomized rats, after the application of a long or short term *in vivo* administration protocol of AMG641, respectively. Our data cannot be easily compared with those of the study by Mendoza et al., [58], due to different *in vivo* versus *in vitro* adopted experimental approaches. Moreover, AMG641 has been reported as having different effects attending to cell types [60]. Further comparisons can be performed with previous studies reporting the effects of other selective CaSR agonists on the proliferation of fetal-adnexa derived MSCs. Our group reported that NPS R-467 stimulates cell proliferation in the same cell lines, eUCM-MSCs [13]. Interestingly, the stimulatory effect of AMG641 on cell proliferation was evident even at the very low tested concentrations (0.01 µM) whereas higher concentrations of NPS R-467 (3µM; [13], [38]) or its precursor NPS R-568 (0.3–1 µM; [35]) were reported in previous studies. The stimulatory effect of NPS R-467 was confirmed in the present proliferation study, as it was used as positive control. This finding could be considered of high importance for the use of this novel potent calcimimetic as a component of media for fetal-derived MSC cultures, in protocols aimed to obtain enough cells for downstream purposes of cell therapy applications.

Given that it was previously demonstrated that physiological changes in [Ca^2+^]_o_ control, via CaSR, the fate of progenitor cells of specific lineages (such as osteogenic, neurogenic, adipogenic and epidermic lineages: see Introduction for references) toward their specific differentiation routes, after testing the dose-response effect of AMG641 on cell proliferation, we wanted to test the effects of this potent research calcimimetic on the differentiation potency of fetal mesenchymal stem cell populations, such as UCM-MSCs, considered as less committed and having wider stemness bounderies than progenitor cells of specific lineages.

In UCM-MSCs, we showed that keeping elevated [Ca^2+^]_o_ in culture medium during osteogenic differentiation induced over-expression of bone differentiation-related genes. The observed cell responses had different extent in the two examined cell lines. In detail, in the large cell line, high [Ca^2+^]_o_ up-regulated OPN mRNA expression, whereas in the small cell line it increased both OPN and RUNX2 mRNA levels. These results may be interpreted in light of different roles played by these osteogenic biomarkers. Indeed, RUNX2 (also known as Core binding factor alfa 1, Cbfa 1) is the master osteoblast transcription factor responsible for osteoblast differentiation and mineralization whereas OPN is a major osteoblast organic matrix protein downstream of RUNX2 [42]. Thus, it could be possible that in the small cell line (less committed), high [Ca^2+^]_o_ was able to up-regulate the master gene RUNX2 and, consequently, its downstream gene OPN. Whereas, in the large cell line (more committed), high [Ca^2+^]_o_ was only able to stimulate OPN expression. These findings are in part in agreement with studies published meanwhile our study was performed [49], [62]. In human bone marrow mesenchymal stromal cells cultured *in vitro*, elevated [Ca^2+^]_o_ was reported to induce over-expression of genes coding for bone-related extracellular matrix proteins, such as OPN, Osteocalcin and bone sialoprotein but it had no effects on RUNX2 [62]. In the same study, the authors reported evidences demonstrating that the signaling pathway between extracellular Ca^2+^and bone morphogenetic protein 2 (BMP-2), a protein essential to maintain bone homeostasis and having a prominent role in fracture healing, involves type L voltage-dependent Ca^2+^channels (L-VDCC) rather/more than CaSR. A subsequent study strengthen the role of CaSR in supporting osteogenic differentiation, as it demonstrated that extracellular calcium promotes osteogenic differentiation in human periodontal ligament stem/progenitor cells and that CaSR and L-VDCC appear to act reciprocally in mediating this process [63]. In ovine amniotic fluid MSCs, increasing [Ca^2+^]_o_ was reported to increase ALP activity [49]. Our data allow to confirm that Ca^2+^ is a potential osteo-inductive triggerer in UCM-MSCs, and that observed difference in the RUNX2 expression between the two cell lines tested in the present study could be related to differences in their developmental and functional stage (commitment level), as previously reported [12], [13]. Similarly, Ca^2+^-induced up-regulation of osteogenic biomarkers was associated with up-regulation of CaSR expression (significant increase in the large cell line and increasing trend in the small cell line), leading us to hypothesize that Ca^2+^-induced stimulation of osteogenic differentiation could be mediated by CaSR.

The addition of AMG641 during osteogenic differentiation induced opposite effects in the two cell line types. In fact, in the large cell line, AMG641 promoted osteogenic differentiation as it significantly increased OPN transcription, ALP activity and cell mineralization level even if it had no effect on RUNX2 expression, as observed in the same cell line upon stimulation with high [Ca^2+^]_o_. Conversely, in the small cell line, AMG641 significantly down-regulated OPN and RUNX2 transcription even though it significantly increased ALP activity and calcium phosphate deposit formation. This apparent discrepancy may be explained considering that the formation of the organic glycoproteic and inorganic mineralized extracellular matrix are distinct events in the sequence leading to bone formation. Mineralization is regulated by a complex arrangement of stimulatory and inhibitory components [42] and AMG641-mediated CaSR stimulation may result in a balance between stimulatory and inhibitory factors involved in calcium phosphate crystal formation and prevention of eccessive mineralization. Our data on the effects of CaSR stimulation on osteogenic differentiation can be compared with those of the recently published study by Di Tomo et al., [49] who reported that the addition of the calcimimetic R-568, a selective CaSR agonist, in ovine amniotic fluid MSCs, increases cell mineralization (as expressed by ALP and Alizarin red S) and this effect is reversed by Calhex 231, a CaSR allosteric inhibitor. The same group confirmed these results in human amniotic fluid MSCs [53]. Our data allow us to confirm that CaSR is involved in the osteogenic differentiation of UCM-MSC because the research calcimimetic AMG641 used in the present study is a selective CaSR activator [59], [60]. The different molecular cell responses observed in large and small cell lines, may be due to their different developmental and functional maturity (different commitment status, as discussed above), including possible differences in the CaSR gene structure, gene expression regulatory mechanisms or signal transduction pathways, as previously hypothesized by Martino et al, [13]. Interestingly, and unlike observed stimulation with high [Ca^2+^]_o_, the calcimimetic AMG641-induced regulation of osteogenic biomarkers expression was inversely related to that of CaSR transcript, which was significant down-regulated in the large cell line where osteogenic differentiation was promoted, but markedly up-regulated in the small cell line, where osteogenesis was inhibited. This finding is not in agreement with the R-568-induced significant incresase of CaSR protein expression reported by Di Tomo et al., [49] in ovine amniotic fluid MSCs. These differences may be due to differences in the two calcimimetics action mechanisms, probably eliciting different signaling pathways, and/or to differences in the commitment status of analyzed cell lines. As a further confirmation to the observed CaSR-mediated effect on eUCM-MSC, we tried to revert the effect observed in the presence of AMG641 by using a CaSR antagonist, NPS2390. As previously observed, NPS2390 is a potent and selective non-competitive group I metabotropic glutamate receptor (mGluR) antagonist [64]. In relation to CaSR's reported high structural homology with mGluR1 [65], NPS2390 has been used as CaSR antagonist in previous studies [13], [66]–[68]. Indeed, in the present study, NPS2390 reversed the effects of AMG641 on OPN mRNA transcription levels, which were down-regulated in the large cell line and up regulated in the small cell line. As well, in both cell lines it significantly reversed the AMG641-induced effect on ALP activity and calcium phosphate deposit formation. Taken together, our data allowed us to demonstrate that AMG641 significantly enhanced osteogenic differentiation efficiency in equine UCM-MSCs. All these findings suggest that Ca^2+^ and the calcimimetic AMG641 promote osteogenic differentiation via CaSR. However, differences in synergic (Ca^2+^) or inverse (AMG641) regulation of CaSR and osteogenic markers mRNA levels, lead us to hypothesize that these two agonists may act through different pathways.

Concerning neurogenic differentiation, we also showed that keeping elevated [Ca^2+^]_o_, during differentiation culture resulted in the over-expression of a specific gene involved in neurogenesis. Indeed, in both cell lines (even with different significance levels in the two cell types), we found over-expression of the gene coding for Nestin, which is an intermediate filament protein associated with immaturity in the nervous system [69]. Nestin has been reported as a prerequisite for the acquisition of the capacity to progress towards the neural lineage [69] and also as a neuronal stem cells marker implied in neuronal differentiation of MSCs [70], [71]. Moreover, expression of GFAP was never found in our trials, indicating that culture conditions examined in the present study are not useful to walk the differentiation road leading to the development of mature astrocytes [69]. These findings are in line with those of a previous study by Vizard et al., [45] in which it was reported that the effects of increased [Ca^2+^]_o_ in mouse late fetal sympathetic neurons in culture enhances axonal growth, thus showing that calcium is involved in the regulation of the growth of neural processes in the central and peripheral nervous system.

The addition of AMG641 during neurogenic differentiation increased CaSR and Nestin mRNA expression in both the large and the small cell line. Our observations of a synergic up-regulation of CaSR and the neurogenic biomarker Nestin mRNA levels, by both CaSR agonists (Ca^2+^and AMG641) and in both cell lines, even associated with non quantitatively relevant effects on Nissl bodies formation and beta III tubulin immunopositivity, support our hypothesis that CaSR is involved in early-stage neurogenic differentiation of UCM-MSCs, as reported in previous studies in other cell systems [72]. The stimulatory effects of the CaSR agonist observed in our study are also in line with those reported by Vizard et al., [45] who showed that activating CaSR in perinatal sympathetic neurons with the calcimimetic NPS R-467 enhances axonal and dendritic growth in culture and this effect is reversed by the selective CaSR antagonist NPS89636 and cannot be obtained in CaSR deficient mice. Nevertheless, the study by Vizard et al. was performed on perinatal neurons [45]. Lack of reproducible reversion effects by NPS2390 on the analyzed neurogenic differentiation biomarkers (Nissl bodies formation and Nestin mRNA levels) was observed. However, the possibility that NPS2390 could reverse expression or activity of other CaSR-induced neurogenic differentiation biomarkers cannot be excluded.

In conclusion, this study demonstrates that CaSR stimulation, by means of the calcimimetic AMG641 in presence of high [Ca^2+^]_o_, increases cell proliferation and increases osteogenic differentiation efficiency and early-stage neurogenic differentiation in UCM-MSCs, a unique fetal adnexa-derived MSC family with components presenting various stemness degree, thus a unique versatile opportunity in future cell therapy strategies. Moreover, our findings showed that CaSR activation may play a fundamental role in selecting specific differentiation checkpoints of these two differentiation routes, as related to cell commitment status, including differences in CaSR gene and/or protein structure and/or function, and used agonists. This CaSR-mediated selection of specific differentiation checkpoints could be of considerable utility in future therapeutical applications. Lastly, our data open the way to the use of the calcimimetic AMG641 in bone and nervous system regenerative medicine.
